# The strength of conspiracy beliefs versus scientific information: the case of COVID 19 preventive behaviours

**DOI:** 10.3389/fpsyg.2024.1325600

**Published:** 2024-04-04

**Authors:** Daniel Pinazo-Calatayud, Sonia Agut-Nieto, Lorena Arahuete, Rosana Peris, Alfonso Barros, Carolina Vázquez-Rodríguez

**Affiliations:** ^1^University of Jaume I, Castelló de La Plana, Spain; ^2^Miguel Hernández University of Elche, Elche, Spain

**Keywords:** COVID-19, implicit theories, conspiracy beliefs, objective information, preventive behaviours

## Abstract

Controlling the spread of COVID-19 requires individuals to adopt preventive behaviours, but conspiracy beliefs about its origin are spreading. The aim of this paper is to better comprehend the strength of conspiracy beliefs versus objective COVID-19 information to predict people’s adherence to protective behaviours (getting vaccinated, being tracked through APPs, and keeping social distance from infected people). Study 1 shows that COVID-19 implicit theories detected in the Pre-study were activated as independent factors that constitute people’s interpretations of the virus origin. These beliefs were related to a lesser intention to engage in preventive behaviours and a higher level of mistrust in institutional information, although some beliefs generate positive expectations about COVID-19 consequences. In Study 2, conducted with a different sample, official COVID-19 information was included as an independent variable, but this new variable did not further explain results. Lastly, Study 3 consisting of both previous samples confirmed that conspiracy beliefs had a direct effect on a lesser willingness to engage in preventive actions, a higher mistrust, and positive expectations about COVID-19 consequences. We conclude that objective COVID-19 information did not buffer the effect of conspiracy beliefs; they interfere with actions to prevent it by taking institutions as scapegoats or complicit with secret powers.

## Pre-study

### Introduction

Every time we interpret an event, we predict someone’s behaviour and make the decision to act in a specific way. This is because we adopt a certain way of “seeing” reality. We are guided by an implicit theory. According to the Implicit Theories Model (Rodrigo et al., 1993), people develop implicit theories (ITs) about the social world through a socio-constructive process. These theories can function as either knowledge (declaratively) or a belief (in an interpretive and prescriptive way). So, despite people possibly knowing a wide variety of explanations about the origin of COVID-19, they only believe some of them, and thus, they interpret reality from there and act accordingly. This dual functionality requires a flexible and dynamic structural model, and knowledge or beliefs can be synthesised depending on demand (Hintzman, 1986). Moreover, depending on the individual motivation, a specific synthesis of beliefs is activated. However, if motivation changes, another different synthesis of beliefs can be activated, which explains intraindividual variability. In addition, the limits of ITs are blurry, since people activate several theories simultaneously in the same context (Rodrigo et al., 1993). The aim of the Pre-study was to explore which ITs people know about the origin of COVID-19 (COVID-19 ITs). To do so, a process was developed that included two phases.

## Conspiracy beliefs

Empirical evidence suggests that the aversive feelings that people experience in crises (i.e., fear, uncertainty, not feeling in control) stimulate a need to control and make sense of the situation, which increases the likelihood of perceiving conspiracies in such social situations (van Prooijen and Douglas, 2017). It is not surprising that conspiracy theories flourished shortly after the first COVID-19 news and still spread (Van Bavel et al., 2020). The disease is not easily explained, it affects people’s lives globally, and uncertainty prevails (Imhoff and Lamberty, 2020). Hence conspiracy beliefs might be potentially palliative in giving individuals back their sense of control (Imhoff and Lamberty, 2020).

Conspiracy beliefs are a group of false ideas in which the ultimate cause of certain events or situations is judged as being a plot devised by many actors working together with a clear goal in mind, which is unlawful and secret (Swami et al., 2014) and with a negative intent (European Commission, 2020). Some conspiracy beliefs are about the origins of the SARS-CoV-2 virus; for example, it is a hoax or an exaggeration of governments, it is a human-manufactured virus (Imhoff and Lamberty, 2020), a bioweapon created by China to destroy the West (Freeman et al., 2020), or electromagnetic waves transmitted by 5G technology (Jolley and Paterson, 2020). Even Jews are the target of conspiracy theories (i.e., Jews control the government, the media, or banks for malicious purposes) (European Commission, 2020). Other theories focus on prevention and cure and state; for instance, conventional medical treatment should not be trusted and people should use alternative remedies to ward off the virus (Van Bavel et al., 2020).

## Conspiracy beliefs and their effects

Conspiracy beliefs can have harmful consequences and might motivate problematic behaviour in the current crisis. They may fuel discrimination, justify hate crimes, and spread mistrust in public institutions, which could lead to political apathy or radicalisation, and even mistrust in scientific and medical information, with very serious consequences (European Commission, 2020). When people are faced with decisions in their lives that involve uncertain or complex knowledge, they tend to rely on institutions to make them (Jost and Hunyady, 2005; Kay et al., 2008; Shepherd and Kay, 2012).

However, conspiracy beliefs may break this tendency to trust in institutional information. Empirical evidence reveals that the people who endorse a conspiracy worldview are not especially likely to trust expert recommendations that aim to lower infection rates (Imhoff and Lamberty, 2020). Conspiracy beliefs connect mistrusting institutions and experts, such as adhering less to all government guidelines, being less willing to undergo diagnostic or antibody tests, or be vaccinated, and are also associated with climate change conspiracy beliefs (Freeman et al., 2020). Holding more COVID-19 conspiracy beliefs is related to adhering less to containment-related behaviours both directly and indirectly by trusting the government, the health system, and their experts less (Karić and Međedović, 2021). Similarly, COVID-19 conspiracy beliefs that either minimise its importance or blame it on actors are presumed to have a malicious intent and are inversely related to both reports of taking preventive actions and intentions to be vaccinated (Romer and Jamieson, 2020). Belief in the efficacy of malicious intervention by the health care system is often coupled with the perception (Eicher and Bangerter, 2015; Taylor and Asmundson, 2020).

Imhoff and Lamberty (2020) explored the idea that different forms of conspiracy beliefs have distinct behavioural implications and found that the distorted beliefs describing the pandemic as a hoax were more closely linked with reduced containment-related behaviour (e.g., hygiene, physical distancing), while conspiracy beliefs in sinister forces purposefully creating the virus were related to more self-centred prepping behaviour (e.g., alternative remedies, hoarding). Along these lines, Bolsen et al. (2020) found that those individuals who believed the virus originated naturally from zoonotic transmission (i.e., from bats to humans) were more supportive of additional funding for biomedical research to identify harmful coronaviruses. However, exposure to conspiracy rhetoric (i.e., SARS-CoV-2 originated in a Chinese laboratory) in isolation, or even competing with scientific information about its natural origin (i.e., a debate between scientists and others about the origin of the virus being shown to participants), resulted in a so-called conspiracy effect. This reduces individuals’ intentions to urgently practice necessary public health behaviours, such as wearing face masks, frequently washing hands, and maintaining a 6-foot social distance. Even the belief in a 5G conspiracy is associated with violent responses to the presumed connection between 5G mobile technology and COVID-19. This relation is explained by state anger, where the effect between anger and violence is stronger for those with heightened paranoia (Jolley and Paterson, 2020).

## The present research

Due to COVID-19’s high contagion, which seems even higher in the new variants of the mutated strain, controlling the spread of this virus requires people adopting preventive behaviours globally. Understanding the factors that predict individuals’ willingness to engage in such preventive actions is essential for controlling infection (Romer and Jamieson, 2020). The argument of the difficulty to understand complex information, in this case the origin of COVID-19, suggests that people tend to rely on institutions to make decisions, which implies that they demand actions (Jost and Hunyady, 2005; Kay et al., 2008; Shepherd and Kay, 2012).

However, previous research has demonstrated that a significant minority of the population holds clear false beliefs of COVID-19 conspiracies (Freeman et al., 2020), which are related to mistrust in scientific, expert, and medical information and recommendations (e.g., European Commission, 2020; Freeman et al., 2020; Imhoff and Lamberty, 2020; Karić and Međedović, 2021). These conspiracy beliefs are also associated with being less willing to be vaccinated (e.g., Freeman et al., 2020; Romer and Jamieson, 2020) or performing fewer containment-related behaviours, such as those related to hygiene or social distancing (Imhoff and Lamberty, 2020). These studies suggest that beliefs operate as knowledge; that is, people do not need to rely on institutions to perform their actions because, even if they do not understand the available information, they are convinced that they understand it and have drawn their own conclusions and know how to act.

In addition to the conspiracy beliefs described in previous studies, further knowledge is required about which ones dominate and how they affect preventive behaviours, including an important protective one; that is, physical distancing from infected people. This is essential because conspiracy beliefs may minimise the perception of risk contagion. Another factor that might affect willingness to engage in protective behaviours is beliefs in the future, i.e., expectations. We argue that when people believe that COVID-19 is due to a plot by one or many actors working together in secret and with the negative intent to somehow control people, only the pandemic’s harmful consequences can be expected and, hence, reinforces their unwillingness to perform protective actions. However as far as we know, its association with preventive behaviours has not yet been tested. Expectations of society’s future have only been studied as a consequence of perceived political polarisation and the perception of a chaotic government response (Crimston and Silvanathan, 2020). Here we attempt to bridge these gaps.

The aim of this research is to extend previous studies by testing which factors, including the endorsement of distinct COVID-19 conspiracy beliefs, are predictive of willingness to engage in three preventive behaviours: getting vaccinated, being tracked through apps, and physical distancing from infected people. To do so, we carried out a pre-study and three subsequent studies, which did not involve medical experimentation and were approved by the Research Ethics Committee of the university to which the main researcher belongs and where the research was conducted. Participation was voluntary. The participants’ consent was obtained when they clicked on the link to start the online survey after being informed about the research purpose. This consent was necessary to complete the survey.

### Method and results

The method and results of this study were differentiated into two phases, which responded to distinct conceptual purposes.

#### Phase 1. Exploratory analysis

This phase had a twofold aim. Firstly, to obtain the different alternative opinions on the origin of COVID-19 using a historical review technique. The compilation of the theories was carried out on Internet channels known to focus on conspiracy theories of different tenors (e.g., Forocoches, Pandora’s box, MindaliaTV, and some others identified in a random search by the research group). All of these channels had discussion forums. Twitter, Facebook, and Instagram were the only social networks used as well as some YouTube accounts. They were chosen for their implicit reputation in terms of their promoting of ideas that could be considered conspiracy theories. An additional criterion was to select channels in the language of the country where the research was conducted and with a main presence in that country. About 74 statements or items (see Supplementary Table S1 for details) were collected in April 2020. The second aim was to classify the different statements according to their similarity and coherence. To this end, a focus group was formed in which five expert academics (three women and two men aged 45–61 years) were asked to classify different items. A final list of 30 items was classified into five cultural categories, with six items per category. The 30 items were used in Phase 2 to develop data collection instrument items (see Supplementary Table S1 for details). Forty-four ambiguous and redundant items were removed (see Supplementary Table S1 for details).

#### Phase 2. Analysis of COVID-19 its as knowledge

The aim of Phase 2 was to determine the structure of COVID-19 ITs as knowledge. We hypothesised that people collected ideas about the origin of COVID-19 (COVID-19 ITs), which they organise prototypically as mental representations. Five questionnaires (one questionnaire per theory) were devised with the 30 items selected in Phase 1. They were similar but with a different cover. On each cover, two individuals had a conversation and defended one of the five theories (for more details, see Supplementary Table S2). A sample of 110 participants (54 men and 56 women aged between 19 and 65 years) agreed to voluntarily collaborate in the study over the Internet. Each participant answered one questionnaire (see an example in Supplementary Table S3) and was asked to respond as the leading characters of the story, after clarifying that these individuals’ opinions were required and not their own, on a 5-point Likert scale ranging from 1 (*Totally disagree*) to 5 (*Totally agree*). The result was compared to the structure obtained from the expert group. The items and structure that matched in both cases were maintained.

In order to understand the relevance of each statement in its theory of belonging, the Typicity Index (Rosch, 1975) was used. It provides the degree of representativeness of elements belonging to a mental representation. The findings were obtained from individuals’ average scores, which showed to what extent each statement was typical of each theory. Typical items were those whose index was 4 or higher (see Supplementary Table S4 for details). According to this criterion, 10 items were removed and the remaining 20 were distributed in four theories. In addition, an exploratory factor analysis (EFA) with principal components extraction was carried out. The Kaiser–Meyer–Olkin (KMO) measure of sampling adequacy and Bartlett’s test of sphericity gave good results: KMO = 0.85, (*χ*^2^ = 1377.934; df = 190; *p* < 0.001). The factorial solution showed four factors (see Supplementary Table S4 for details).

### Discussion

The aim of the Pre-study was to detect which COVID-19 origin theories people knew and whether they were organised as mental representations, following previous research that refers to knowledge of the world being organised as ITs (Hintzman, 1986; Rodrigo et al., 1993). As we expected, ideas about COVID-19 origin theories were prototypically organised as mental representations (COVID-19 ITs). Twenty items were organised into four mental representations that people activated as declarative knowledge synthesis when asked about their COVID-19 origin knowledge. Those items composed the COVID-19 ITs questionnaire that we used in subsequent studies.

## Study 1

In Study 1, we expected these theories to function as conspiracy beliefs linked with mistrust in institutional information, negative expectations of the pandemic’s consequences, and being less willing to engage in preventive behaviours. The aim of this study was to: (1) analyse the relationships among those four theories, activated as conspiracy beliefs, and the intention to engage in three preventive behaviours (getting vaccinated, being tracked through apps, and physical distancing from infected people); (2) compare the hypothesis of direct relations (beliefs as factors that directly affect preventive behaviours and also mistrust in institutional information and expectations of the pandemic’s consequences) to the hypothesis of difficulty in understanding information (i.e., this will imply a direct effect of conspiracy beliefs on preventive behaviours, as well as the mediation of mistrust and expectations of this relation).

### Method

We examined whether the four mental representations were activated as conspiracy beliefs also grouped into independent factors. We also analysed whether conspiracy beliefs were linked with being less willing to engage in protective behaviours by testing two models: M1, which proposed a direct relation; M2, which proposed a partial mediation of expectations and mistrust. Accordingly, we posed three hypotheses:

*H1*: Knowledge theories about the origin of COVID-19 would be activated as independent factors of conspiracy beliefs.

*H2*: Conspiracy beliefs would be related to less intention to engage in preventive behaviours, worse expectations of COVID-19 consequences, and more mistrust in institutional information (M1: direct relation).

**Figure 1:**
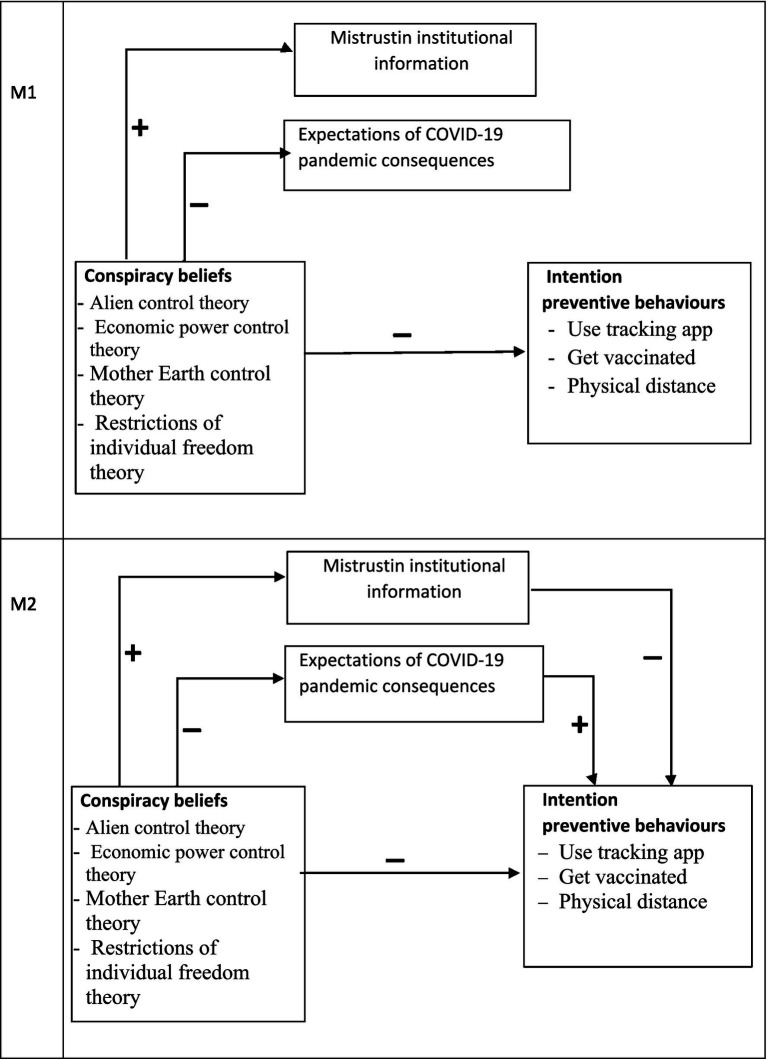
Proposed direct relation model (M1) and proposed partial mediation model (M2), Study 1. *N* = 265.

*H3*: Conspiracy beliefs would be related to being less willing to engage in preventive behaviours, having fewer positive expectations of the pandemic’s consequences, and more mistrust. In turn, mistrust would be related to fewer preventive behavioural intentions, while more positive expectations would be related to being more willing to engage in preventive behaviours (M2: partial mediation) (see Figure 1).

#### Sample

The sample contained 265 participants (89 male, 176 female). Their mean age was 45.39 years (SD = 12.86), within the 18–78 years range. Of the whole sample, 67.2% had at least a university degree while the remaining 32.8% did not. As far as we know, there is no way to estimate sample size effects in structural equation models.

#### Procedure

The participants completed an online survey to assess the relations between conspiracy beliefs and willingness to engage in protective behaviours “for COVID-19”, must apply in the Methods section of Study 2. This survey was distributed to people on social media platforms (e.g., Facebook, WhatsApp, YouTube, e-mail, etc.).

#### Variables

##### Conspiracy belief theories about the origin of COVID-19 (COVID-19 ITs)

To assess the conspiracy belief theories about the origin of COVID-19, we used the COVID-19 ITs questionnaire developed in the Pre-study (see Supplementary Table S5 for details). The wording of items was modified to make them self-reflective. This allowed us to change the Pre-study items that deal with knowledge (e.g., “COVID-19 has been created by laboratories to sell drugs”) to items that assess beliefs (e.g., “I believe coronavirus has been created by laboratories to sell medicines”) (see Supplementary Table S6 for details). An examination of the Kaiser–Meyer–Olkin measure of sampling adequacy suggested that the sample was factorable (KMO = 0.899). An EFA with principal components extraction was carried out for this sample. The four factors obtained by varimax rotation, by choosing those components with eigenvalues above 1, were similar to those in the Pre-study and explained 63.61% of the variance. The first factor, *Alien CT*, was composed of five items (eigenvalue = 7.71). The item with the highest factor load (*r*^2^ = 0.90) was: “I believe COVID-19 vaccine is programmed by aliens to subdue us.” The second factor, *Economy CT*, included four items (eigenvalue = 2.06). The item with the highest factor load (*r*^2^ = 0.82) was: “I believe coronavirus has been created by laboratories to sell drugs.” The third factor, *Earth CT*, comprised six items (eigenvalue = 1.77). The item with the highest factor load (*r*^2^ = 0.78) was: “I believe coronavirus has been caused by nature itself to humanity.” Lastly, the fourth factor, *Freedom restriction T*, was composed of five items (eigenvalue = 1.19). The item with the highest factor load (*r*^2^ = 0.78) was: “I believe COVID-19’s tracking apps have been created to control people.”

##### Mistrusting institutional information (Mistrust)

To assess the degree of mistrust in the information provided by institutions, mass media, etc., to control the pandemic, we used a 4-item questionnaire devised for this study (e.g., “I feel manipulated”) (see Supplementary Table S6 for details). The participants answered on a 5-point Likert scale ranging from 1 (*Totally disagree*) to 5 (*Totally agree*). Higher scores were indicative of more mistrust.

##### Expectations of the pandemic’s consequences (Expectations)

To evaluate people’s expectations of the SARS-CoV-2 pandemic’s consequences, we used a 7-item questionnaire designed for this study. Four of the items described expectations of positive pandemic consequences (e.g., “Scientific thinking will be strengthened”) (see Supplementary Table S6 for details). The remaining three items expressed negative consequences (e.g., “Our civilisation as we know it will collapse”) (see Supplementary Table S6 for details). These last items were reversed so that higher scores would be indicative of more positive expectations and vice versa. The participants responded on a 5-point Likert scale ranging from 1 (*Totally disagree*) to 5 (*Totally agree*).

##### Intention to install a COVID-19 tracking apps (Apps)

To assess people’s willingness to engage in installing a tracking app, we used a self-devised questionnaire with five items (e.g., “I will use a tracking app to find out if I am near someone infected”) (see Supplementary Table S6 for details). The participants replied on a 5-point Likert scale ranging from 1 (*Totally disagree*) to 5 (*Totally agree*). Higher scores were indicative of more willingness to accept a COVID-19 tracking app.

##### Intention to get vaccinated (Vaccine)

People’s behavioural intention to get vaccinated was evaluated by using a self-devised questionnaire with two items: “I will get vaccinated as soon as there is a vaccine available” and “I am not going to get vaccinated” (reverse) (see Supplementary Table S6 for details). The participants answered on a 5-point Likert scale ranging from 1 (*Totally disagree*) to 5 (*Totally agree*). Higher scores were indicative of being more willing to get vaccinated.

##### Physical distancing from infected people (Distancing)

To assess the extent to which people are willing to be physically distanced from infected people, we adapted the “Fear and avoidance” subscale from the Community Attitudes towards Mental Illness Scale (CAMI-S; Taylor and Dear, 1981; Högberg et al., 2008). For the purpose of this study, we changed the topic of serious mental illness to physical distancing from people infected with COVID-19 as a protective measure and not due to stigmatisation. We argued that the people with conspiracy beliefs would not accept the existence or danger of COVID-19 and would be likely to reject this physical distancing because they would not perceive being at risk from the infected population when constituting recommended protective behaviour. The 4-item questionnaire was: “It is best to avoid anyone who has tested positive for COVID-19” (see Supplementary Table S6 for details). The participants answered on a 5-point Likert scale ranging from 1 (*Totally disagree*) to 5 (*Totally agree*). Higher scores were indicative of them being more willing to physically distance from infected people.

### Results

Table 1 displays the descriptive analyses and correlations of this study. Data reveal our participants’ low adhesion level to conspiracy beliefs about the origin of COVID-19.

**Table 1:** Descriptive analysis and correlations of the variables in Study 1 (*N* = 265).

	M	SD	*α*	2	3	4	5	6	7	8	9
1. Alien CT	1.28	0.59	0.81	0.511^**^	522^**^	0.453^**^	0.338^**^	0.344^**^	−0.274^**^	−0.419^**^	−0.054
2. Economy CT	2.09	1.11	0.88	1	525^**^	0.623^**^	0.617^**^	0.412^**^	−0.385^**^	−0.325^**^	−0.045
3. Earth CT	2.24	0.89	0.81		1	0.425^**^	0.298^**^	0.579^**^	−0.244^**^	−0.226^**^	0.007
4. Freedom RT	2.75	1.02	0.79			1	0.647^**^	0.398^**^	−0.457^**^	−0.305^**^	−0.125^*^
5. Mistrust	3.40	1.05	0.79				1	0.307^**^	−0.384^**^	−0.230^**^	−0.095
6. Expectations	2.96	0.67	0.67					1	−0.220^**^	−0.180^*^	0.030
7. Apps	3.14	1.01	0.78						1	0.472^**^	0.199^**^
8. Vaccine	3.74	1.37	0.83							1	0.213^**^
9. Distancing	2.13	1.00	0.71								1

^*^p < 0.05 and ^**^p < 0.01.

**Figure 2:**
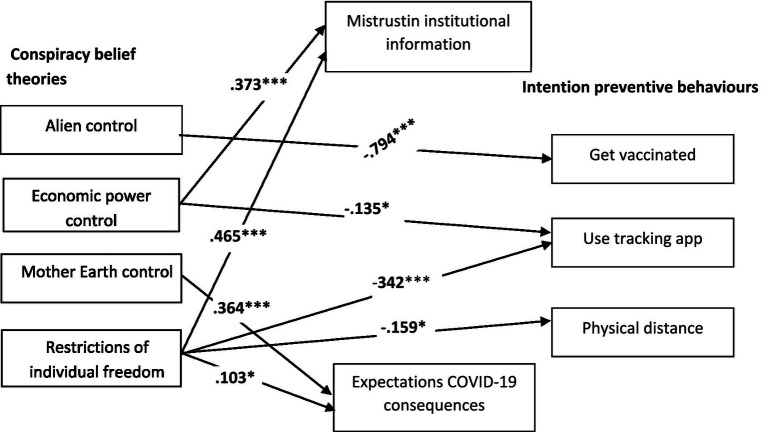
Final estimated model (M1), Study 1. *N* = 265. Only the significant standardised path coefficients are provided; ^*^p < 0.05 and ^***^p < 0.001.

To test the hypotheses, two plausible models (see Figure 1) were compared by following the maximum likelihood estimation method of structural equation modelling (SEM), as implemented by the AMOS 26 computer program (Arbuckle, 2019). The first model proposes a direct relation of conspiracy beliefs to preventive behavioural intentions, as well as mistrust and expectations. M1 fitted the data well (*χ*^2^ = 4.475; df = 7; *p* = 0.724; RMSEA = 0.000; NFI = 0.995; CFI = 1.000). The second model proposes a partial mediation of mistrust and expectations of the relation between conspiracy beliefs and behavioural intentions. M2 also fitted the data well (*χ*^2^ = 0.521; df = 1; *p* = 0.470; RMSEA = 0.000; NFI = −999; CFI = 1.000). Although both models fitted the data well, the comparison between them was favourable for the first one (RFI_ME1_ = 0.972 vs. RFI_ME2_ = 0.978). A parsimonious and comparative index was calculated, which also revealed that M1 was better than M2 (AIC_M1_ = 80.475 vs. AIC_M2_ = 88.521). The final model (M1) is depicted in Figure 2.

### Discussion

In Study 1, we explored whether COVID-19 ITs would be activated as conspiracy beliefs and how they would affect the population’s mistrust, expectations, and willingness to be involved in behaviours to prevent infection. Firstly, as expected in H1, the results revealed that the COVID-19 ITs about its origin were activated as independent factors to constitute people’s interpretations of the SARS-COV-2 origin. In addition, the findings revealed that M1 fitted the data better than M2, and it also explained the relations between the variables with fewer estimators. Therefore, H3 was not supported. H2, which predicted a direct effect of COVID-19 ITs on behavioural intentions and mistrust, was confirmed, but not for expectations. Therefore, H2 was partially confirmed. To better clarify these relations, Study 2 aimed to replicate these results and compare them to a model that involved institutional information to explain commitment to preventive behaviours, as well as expectations and mistrust.

## Study 2

Study 2 incorporated the variable official COVID-19 information. We expected that, according to the degree to which people have official information about the virus, its severity, and possible consequences, they would perceive the future to be under control (more positive expectations) and would be more predisposed to perform protective behaviours. However, they would likely mistrust institutional information and conclude that the whole matrix of official power and powers behind it would likely provide a partial or biased vision of the pandemic. In parallel, other individuals could develop conspiracy beliefs that could result in a false sensation of being informed. In fact, previous research reveals that believers in conspiracy theories are news consumers and feel informed, but they are nourished by sources not legitimised by official power (Stempel et al., 2007; Klein et al., 2019). This perception of being informed makes people less dependent on the government and, consequently, they trust institutions and their actions less. They might hold a more pessimistic vision of the future (generating more negative expectations of the pandemic’s consequences). Study 2 aimed to replicate the Study 1 results with a different sample and to compare M1 to another model that included an additional independent variable: official COVID-19 information (M3). We argued that if official information was included in the model, this variable would have a significant direct effect and M3 would better fit the data than M1 whenever information was not included.

### Method

**Figure 3:**
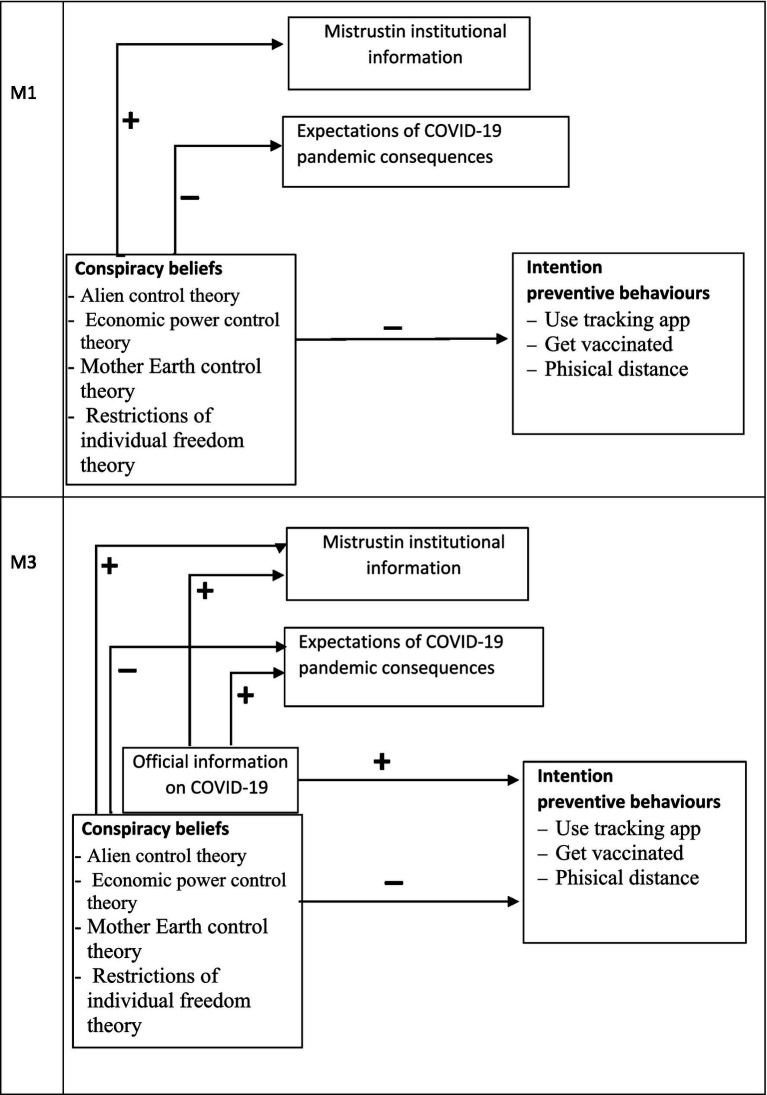
Proposed direct relation model (M1) and proposed direct relation model including official COVID-19 information (M3), Study 2. *N* = 142.

In this study, we tested the effect of official COVID-19 information on mistrust, expectations, and willingness to engage in protective behaviour for COVID-19 contention. To do so, we tested two models: M1, that proposed a direct relation, and M3, which included official information (see Figure 3). Accordingly, we posed an additional hypothesis:

*H4*: Official COVID-19 information would be related to more intention to engage in preventive behaviours, mistrusting institutional information more, and more positive expectations of the pandemic’s consequences (M3: direct relation including official COVID-19 information).

#### Sample

This study included 148 individuals, but six of them did not answer the questions about official COVID-19 information so they were removed from the study. The final sample included 142 participants (37 male, 105 female). Their mean age was 43.50 years (SD = 11.33), which fell within the 18–82 years range. Of our sample, 71.8% had at least a university degree while the remaining 28.2% did not.

#### Procedure

Two months after the Study 1 data collection, a different sample of participants completed the online survey, which included the scale about official COVID-19 information. Once again, the survey was distributed through social media platforms (e.g., Facebook, WhatsApp, YouTube, e-mail, etc.).

#### Variables

We employed the same survey that we distributed in Study 1, except for the official COVID-19 information variable. We conducted EFA for this sample of the COVID-19 ITs.

##### Official COVID-19 information

A questionnaire that assessed the official information that people have about this virus was developed by the authors according to common clinical and community COVID-19 management guidelines of the Spanish Ministry of Health. This questionnaire contained 20 items, of which the formulation of nine was false (see Supplementary Table S6 for details). These items were answered on a 5-point Likert scale ranging from 1 “Completely disagree” to 5 “Completely agree.” The right answer rates of the 20 questions in the COVID-19 information questionnaire were 31–97.2%. The mean COVID-19 information score was 79.4% (SD = 14.3; range: 10–100%) in this information test.

##### COVID-19 ITs

These are the conspiracy belief theories about the origin of COVID-19. This sample was also factorable (KMO = 0.822). The EFA with principal components (varimax rotation) for this sample replicated the same four factors and explained 60.23% of the variance: *Alien CT* (eigenvalue = 6.93), *Economy CT* (eigenvalue = 2.25), *Earth CT* (eigenvalue = 1.74), and lastly *Freedom restriction T* (eigenvalue = 1.3). The Cronbach alphas of all the variables appear in Table 2.

**Table 2:** Descriptive analysis and correlations of the variables in Study 2 (*N* = 142).

	M	SD	*α*	2	3	4	5	6	7	8	9	10
1. Alien CT	1.43	0.52	0.83	0.473^**^	0.453^**^	0.338^**^	0.264^**^	0.251^**^	−0.135	−0.359^**^	−0.021	−0.206^*^
2. Economy CT	1.08	0.94	0.88	1	0.525^**^	0.623^**^	0.617^**^	0.412^**^	−0.174^*^	−0.240^**^	−0.078	−0.190^*^
3. Earth CT	2.04	0.77	0.80		1	0.425^**^	0.298^**^	0.579^**^	0.113	−0.119	−0.042	−0.100
4. Freedom RT	2.44	0.52	0.77			1	0.647^**^	0.398^**^	−0.364^**^	−0.242^*^	−0.009	−0.240^**^
5. Mistrust	2.89	0.94	0.71				1	0.307^**^	−0.271^**^	−0.152	−0.029	−0.153
6. Expectations	2.78	0.71	0.73					1	0.057	0.012	−0.014	−0.057
7. Apps	3.30	0.93	0.75						1	0.297^**^	0.112	0.178^*^
8. Vaccine	3.83	1.15	0.82							1	0.126^*^	0.143
9. Distancing	2.69	0.82	0.52								1	−0.012
10. Information	79.4	14.3	0.62									1

^*^p < 0.05 and ^**^p < 0.01.

### Results

In Study 2, the descriptive analyses (see Table 2) also revealed the participants’ slight adhesion to conspiracy beliefs of the origin of COVID-19 and moderate correlations between some variables.

**Figure 4:**
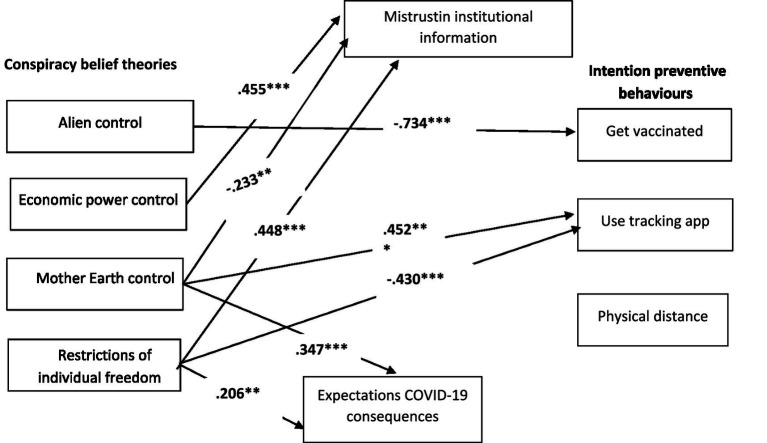
Final estimated model (M1), Study 2. *N* = 142. Only the significant standardised path coefficients are provided; ^**^p < 0.01 and ^***^p < 0.001.

To test H4, M1 and M3 (see Figure 3) were compared by following the maximum likelihood estimation method of structural equation modelling (SEM), as implemented by the AMOS 26 computer program (Arbuckle, 2019). M1 fitted the data well (*χ*^2^ = 7.446; df = 7; *p* = 0.384; RMSEA = 0.021; NFI = 0.981; CFI = 0.999). M3, which included official COVID-19 information, also fitted the data well (*χ*^2^ = 7.110; df = 7; *p* = 0.418; RMSEA = 0.011; NFI = −0.983; CFI = 1.000). Despite both models fitting the data, the comparison of both favoured M1 (RFI_ME1_ = 0.903 vs. RFI_ME3_ = 0.889). Moreover, the parsimonious and comparative index was also calculated and, once again, revealed that M1 was better than M3 (AIC_ME1_ = 83.446 vs. AIC_ME3_ = 103.110). The final estimated model (M1) is displayed in Figure 4.

### Discussion

Study 2 tested whether official COVID-19 information reduced or eliminated the negative effect of conspiracy beliefs on willingness to engage in preventive behaviour for COVID-19. We also aimed to further understand how mistrust and expectations of the pandemic’s consequences would be affected by this new independent variable. In M3, we noted that official COVID-19 information had no path coefficient to indicate a significant effect on either mistrust and expectations, or willingness to engage in preventive behaviours. So, the participation of this variable in the model was spurious and, therefore, H4 was not supported. Whenever the variable official COVID-19 information was not included, M1 displayed better relative fit indices and was more parsimonious.

## Study 3

Study 2 evidenced that the different conspiracy beliefs had distinct direct relations with the dependent variables. The aim of Study 3 was to better clarify the results by confirming M1 with an aggregate sample (the participants jointly from Study 1 and Study 2).

### Method

#### Sample

The sample comprised 407 participants (126 male, 281 female). Their mean age was 44.73 years (SD = 12.37), which fell within the 18–82 years range. Of our sample, 68.8% had at least a college degree while the remaining 31.2% did not.

#### Procedure and variables

The procedure and the variables in Study 3 were the same as those employed in Study 1 and Study 2. This sample was also factorable (KMO = 0.912). The EFA with principal components (varimax rotation) for this sample replicated the same four factors and explained 60.23% of the variance: *Alien CT* (eigenvalue = 7.54), *Economy CT* (eigenvalue = 2.07), *Earth CT* (eigenvalue = 1.71), and lastly *Freedom restriction T* (eigenvalue = 1.10). The Cronbach alphas of all the variables appear in Table 3.

**Table 3:** Descriptive analysis and correlations of the variables in Study 3 (*N* = 407).

	M	SD	*α*	2	3	4	5	6	7	8	9
1. Alien CT	1.34	0.57	0.841	0.476^**^	0.481^**^	0.392^**^	304^**^	0.292^**^	−0.220	−0.388^**^	−0.009
2. Economy CT	1.99	1.06	0.871	1	0.540^**^	0.625^**^	0.617^**^	0.423^**^	−0.328^*^	0.300^**^	−0.086
3. Earth CT	2.17	0.86	0.804		1	0.425^**^	0.283^**^	0.558^**^	−0.144^**^	194^**^	−0.037
4. Freedom RT	2.64	1.00	0.778			1	0.647^**^	0.425^**^	−0.433^**^	0.282^*^	−0.127^*^
5. Mistrust	2.98	1.02	0.770				1	0.303^**^	−0.353^**^	0.209^**^	−0.093
6. Expectations	2.90	0.69	0.696					1	−0.132^**^	0.117^*^	−0.018
7. Apps	3.20	0.98	0.774						1	−400^**^	0.197^**^
8. Vaccine	2.21	1.29	0.831							1	−0.183^**^
9. Distancing	2.32	0.98	0.680								1

^*^p < 0.05 and ^**^p < 0.01.

### Results

The descriptive results of Study 3 are displayed in Table 3. As previously mentioned, the participants obtained low scores for conspiracy beliefs about the origin of COVID-19 but correlated negatively with preventive behavioural intentions.

**Figure 5:**
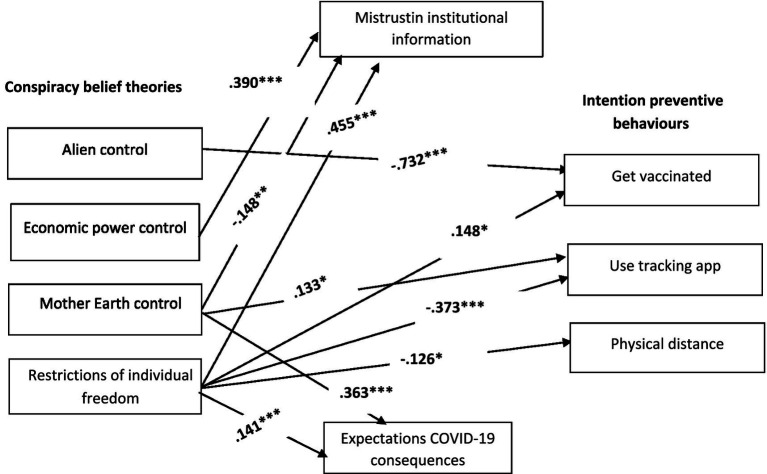
Final estimated model (M1), Study 3. *N* = 407. The significant standardised path coefficients are provided; ^**^p < 0.01 and ^***^p < 0.001.

We tested M1 (see Figure 1) using the entire sample that included the participants from Study 1 and Study 2. As we can see in Figure 5, the findings revealed that this model fitted the data well (*χ*^2^ = 4.771; df = 7; *p* = 0.688; RMSEA = 0.000; NFI = −0.996; RFI = 0.980; CFI = 1.000).

### Discussion

Study 3 aimed to confirm M1 by integrating the two previous studies. The results supported M1 because conspiracy beliefs generally had a direct effect on willingness to engage in preventive behaviours, mistrust, and COVID-19 expectations.

## General discussion

The purpose of this paper was to further understand the strength of conspiracy beliefs versus objective COVID-19 information to predict people’s adherence to behaviours to prevent SARS-CoV-2 from spreading. To do so, we tested and compared three adjustment models. In Study 1, we compared H1, which proposed that conspiracy beliefs would have a direct effect on mistrusting institutional information, expectations of the pandemic’s consequences, and preventive behavioural intentions (M1) with H2, which predicted a mediation effect of mistrust and expectations of the relation between conspiracy beliefs and intention to engage in preventive actions (M2). In Study 2, we also compared H1 to an additional hypothesis (H3), which included official COVID-19 information as an independent variable, to see whether it would buffer the effect of conspiracy beliefs on behavioural intentions (M3). Lastly, Study 3, which included both the previous samples, confirmed that conspiracy beliefs had a direct effect on willingness to engage in preventive actions with neither the indirect intervention of mistrust nor expectations of COVID-19 consequences and, more interestingly, without the participation of official COVID-19 information.

Our findings indicated important advances compared to previous research. It adds a relevant finding about the distinctive and noteworthy relations of conspiracy beliefs in individual freedom restrictions. Therefore, despite some messages stressing the benefits of following COVID-19 health instructions increasing willingness to engage in these actions (Jordan et al., 2020), conspiracy beliefs seem to interfere with these messages by inhibiting possible engagement with prevention actions. Our results support recent studies about the negative influence of conspiracy beliefs on attitudes and behaviours in relation to COVID-19 contention (e.g., Bolsen et al., 2020; Freeman et al., 2020; Imhoff and Lamberty, 2020; Romer and Jamieson, 2020; Karić and Međedović, 2021). On the one hand, our findings support and complement Shepherd and Kay’s (2012) research about how people trust institutions when a topic is unfamiliar or unknowledgeable. Our study did not reveal that people were more likely to listen and trust the government and status quo and their actions when faced with an unfamiliar issue like the origin of COVID-19; instead, conspiracy beliefs give the impression of understanding unfamiliar information. Therefore, this perpetuates ignorance in a way that implies more mistrust rather than more trust in relation to institutions. Another differential aspect of our study was the origin of the used beliefs. Based on the Implicit Theories Model (Rodrigo et al., 1993), here the beliefs that people state are empirically developed using a socio-constructive process. Lastly, and as far as we know, our study is the first to provide joint evidence that beliefs condition the intention to prevent risk and, regardless of the expected consequences, trusting institutions or available official information.

According to Imhoff and Lamberty (2020), the different forms of conspiracy beliefs have distinct behavioural implications. The conspiracy belief in restrictions of individual freedom is that which most interferes with pandemic control management. Believing in a hidden confabulation to restrict individual freedom would decrease the perception of virus severity. This perception is noted insofar as these individuals are unwilling to maintain physical distance from people with COVID-19 because they assume that contact contagion is not a risky option. From this viewpoint, it is easier to understand that this belief has a negative effect on both government trust in pandemic management and some prevention measures. In particular, this belief negatively affects the preventive actions that limit individuals (i.e., a COVID-19 tracking app perceived as a means to control and disrespect privacy and keeping physical distance from people are judged as limitations of social interactions). However, the positive relation between believing in the individual freedom restriction and the intention of getting vaccinated are seen as protective measures, like other seasonal vaccines for widespread use, which are of free choice. This belief in a plot to restrict individual freedom confers certain optimism to people and the feeling that they control their lives. This feeling of control would the individuals who hold this belief have more positive expectations of the pandemic’s consequences. Gupta et al. (2021) indicate the effect of culture on accepting technological prevention measures. These effects might be stronger in countries with a higher incidence of individualistic culture.

The conspiracy beliefs about restricting freedom contrast with those that Mother Earth is developing an energy change that will affect human consciousness. The people who assume these beliefs are equally optimistic about the future, but unlike believers in a conspiracy to restrict freedoms, believers in the energy control of the Earth rely on institutional management and would, therefore, accept the control of tracking apps. Beliefs in global economic control negatively affect institutional trust but not risk prevention demands. However, beliefs in alien control imply less intention to get vaccinated. In short, beliefs that attribute the origin of the virus to the control of dark forces, regardless of it being economic or alien, would be the most likely to inhibit proactive behaviours to COVID-19 contention.

These beliefs make such powers accountable for the origin of the virus by attributing the intention to manipulate people to them. This attribution may explain the fact that individuals who hold these beliefs do not consider prevention behaviours and even refuse any of them (e.g., do not get vaccinated if the virus is of alien origin). Identifying a culprit would explain mistrusting the government and prevention behaviours (Shariff et al., 2014; Levin et al., 2016). Moreover, believing that an economic power is responsible would make the endogroup/exogroup relation salient. When guilt is attributed to the exogroup, social emotion is anger and the trend of offensive action can be seen more (Yzerbyt et al., 2009). In this case, the tendency to perform offensive action involves ignoring prevention measures and not helping to make them more powerful.

The conspiracy beliefs we have herein worked on do not strictly correspond to the content used in the literature (Sunstein and Vermeule, 2009; Lewandowsky et al., 2013) because at least two of these beliefs (Alien CT and Earth CT) do not refer to human power. However, these beliefs share the irrationality of attributing the origin to hidden powers which manipulate human being’s reasoning and emotion. Implicit beliefs tend to perceive a world in which COVID-19 is an instrument to divert attention from the control that these powers seek to exercise. Beliefs are modulable, and the theories that emerge from them can be subject to change and expansion. It would be necessary to replicate the present study’s findings, considering the current social context and currently emerging conspiracy theories. A more robust pre-study that would allow for a broader range of conspiracy theories to be compiled would be useful in the future. In addition, longitudinal studies could also be carried out, selecting participants based on their adherence to certain beliefs and following their development over time.

In conclusion, the conspiracy beliefs studied herein seem to act as a frame that provides an interpretive narrative of reality that serves, on the one hand, to deal with the SARS-CoV-2 threat but, on the other hand, to also interfere with actions to prevent it by perceiving institutions as scapegoats or accomplices in hidden powers. These beliefs compete with rational or scientific information as alternative narratives. They play an interpretative and prescriptive role that explains mistrust in the institutional version and the inhibition of the scientific information effect (van der Linden et al., 2017; Bolsen and Druckman, 2018). In short, the present findings contribute to theoretical knowledge about how, why, and for whom corrections effectively update misconceptions of controversial topics (Trevors and Duffy, 2020). The study also shows that not all conspiracy or irrational beliefs have negative effects, or the same effects, on COVID-19 risk prevention demands.

### Limitations of the study

The limitations of this study are fundamentally related to the sample and the time space for collecting data. Sample collection was carried out at the beginning of the pandemic during the so-called first wave. At that time, conspiracy theories were less developed, which is reflected in participants’ slight adhesion to those beliefs. For this reason, it would be appropriate to work with balanced samples between believers and non-believers, deniers and non-deniers.

Given that this was a global pandemic situation, something that had not been experienced by society for over a century, this research can show the initial reactions and the mechanisms underlying the reactions. Although the data are relatively outdated, we believe that the mechanism of attributing false beliefs and mistrust of information from reliable institutional sources is still in place. The development of false beliefs justified by a conspiracy theory can be repeated when similar social situations arise. Future research should conduct cross-cultural studies to increase the cultural diversity of the sample.

On the other hand, this research would be completed with a study conducted during the third or fourth wave by analysing whether pandemic exhaustion and impotence in this situation have entrenched, maintained, or modified beliefs and their attributions by contemplating the role of context (cultural variables and intergroup salience).

Nevertheless, the fact that the sample was collected at the beginning of the pandemic allowed us to assess how even slight adherence to irrational beliefs favours mistrust and lack of commitment to actions. This suggests that the influence of these beliefs on people’s cognition is constant once they appear. The sample should also be enlarged to perform an intercultural analysis to know whether the idiosyncrasies of different countries and cultures affect the activation of one IT or other ITs, condition how government pandemic management is perceived, and adherence to preventive behaviours.

In short, a bigger sample size and the inclusion of more variables would help us to further understand how our beliefs in COVID-19 influence our subsequent judgements and behaviours.

The time period in which the preliminary study and the study took place corresponds to the first wave of SARS-CoV-2 infection in 2020. Given that this was a global pandemic situation, something that had not been experienced by society for over a century, this research can show the initial reactions and the mechanisms underlying the reactions. Although the data are relatively outdated, we believe that the mechanism of attributing false beliefs and mistrust of information from reliable institutional sources is still in place. The development of false beliefs justified by a conspiracy theory can be repeated when similar social situations arise.

## Data availability statement

The raw data supporting the conclusions of this article will be made available by the authors, without undue reservation.

## Ethics statement

The studies involving humans were approved by the Research Ethics and Integrity Committee of Vicerrectorate of Research Jaume I University of Castellón [CD/45/2019 Generalitat Valenciana]. The studies were conducted in accordance with the local legislation and institutional requirements. The participants provided their written informed consent to participate in this study.

## Author contributions

DP-C: Conceptualization, Formal analysis, Funding acquisition, Investigation, Methodology, Supervision, Validation, Visualization, Writing – original draft, Writing – review & editing. SA-N: Data curation, Investigation, Methodology, Writing – review & editing. LA: Data curation, Formal analysis, Investigation, Methodology, Writing – review & editing. RP: Formal analysis, Methodology, Writing – original draft. AB: Formal analysis, Methodology, Writing – original draft. CV-R: Formal analysis, Investigation, Supervision, Validation, Visualization, Writing – original draft, Writing – review & editing.
