# Supplementary material for: The strength of conspiracy beliefs versus scientific information: the case of COVID 19 preventive behaviours
Source: Front Psychol. 2024 Apr 4;15:1325600. doi: 10.3389/fpsyg.2024.1325600 (PMC11024355; doi:10.3389/fpsyg.2024.1325600)
Supplement: Supplementary file 1 [file Data_Sheet_1.pdf]

Table_1_SuppInfo

PRE-STUDY. Items selected in Phases 1 and 2 of the Implicit Theories (ITs) research process on the origin of COVID-19.

**Phase 1.<br>Exploratory Analysis**		**Phase 2<br>Analysis of COVID-19 Implicit Theories (Its) as Knowledge**
**Step 1.** ***Historical Review:*** **Items obtained**	**Step 2.** ***Focus group:*** **Items of the Cultural Categories**	**Items of the ITs bout the origin of COVID-19 as knowledge**
1. COVID-19's tracking APPS have been created to control people	*	*
2. The decentralized governance system in Spain is to blame for the inefficient control of COVID-19's spread.		
3. The planet has created COVID-19 to facilitate the development of a higher state of human consciousness.	*	*
4. The Dark Energies want to use COVID-19 to enslave humanity.		
5. The virus will force a debate between authoritarian vs. cooperative-democratic governance systems.		
6. On October 11, 2020, a meteorite with COVID-19 particles fell in the area where the pandemic later originated.	*	
7. The management of the pandemic will show the inability of current policies to solve problems within the Capitalist Model.	*	
8. The Earth has reacted. Humans are destroying it. Hence, it has sent a virus to bring humanity to a standstill.	*	*
9. The virus will spotlight current social inequalities on managing the economic crisis.	*	
10. The coronavirus tracking APPS have been created by an "Intelligence" from another planet.	*	*
11. The coronavirus is one of the effects of 5G towers.		
12. The problem is not COVID-19. It is the socio-economic crisis that will come about due to the precariousness of the system	*	

Phase 1. Exploratory Analysis		Phase 2 Analysis of COVID-19 Implicit Theories (Its) as Knowledge
**Step 1.** ***Historical Review:*** **Items obtained**	**Step 2.** ***Focus group:*** **Items of the Cultural Categories**	**Items of the ITs bout the origin of COVID-19 as knowledge**
13. Since we have not learned by love, the planet makes us learn by suffering.	*	*
14. COVID-19 vaccine is an excuse for an alien power aiming to subdue us.		
15. COVID-19 has been brought about by aliens through 5G.	*	*
16. I am willing to participate in a worldwide meditation to raise the vibration of the planet and kill the virus with love		
17. The coronavirus has been created by laboratories to sell drugs.	*	*
18. COVID-19 is the product of "dark energies" aiming to dominate humanity.		
19. The prophecies announced it: confinement due to the pandemic is the path chosen by the planet to transform human consciousness.	*	*
20. The same person who thinks the earth is overpopulated wants to 'save' your life with a coronavirus vaccine.	*	
21. COVID-19 vaccine will be an opportunity to incorporate a tracking chip to controls us.	*	*
22. The coronavirus has an extra-terrestrial origin.	*	*
23. The COVID-19 crisis jeopardizes democratic freedoms.	*	*
24. Governments want to take advantage of the coronavirus to increase control of the population.		
25. The Earth breathes at last; the environment is being cleaned.		
26. The coronavirus tracking APPS are intended to implicitly violate people's right to privacy.	*	*
27. The coronavirus was conceived as a selective epidemic devised by capitalism to kill the elderly.	*	
28. COVID-19 is the product of an American conspiracy against the Chinese economy.		

Phase 1. Exploratory Analysis — Step 1. *Historical Review:* Items obtained	Phase 1. Exploratory Analysis — Step 2. *Focus group:* Items of the Cultural Categories	Phase 2 Analysis of COVID-19 Implicit Theories (Its) as Knowledge — Items of the ITs bout the origin of COVID-19 as knowledge
29. The worst quarantine for COVID-19 is that of your mind.		
30. COVID-19 vaccine is an excuse from a secretive power aiming to control us.		
31. COVID-19 is the product of a Chinese conspiracy to have world economic power.	*	*
32. The coronavirus has gotten out of control due to political fragmentation.		
33. Quarantine is unnecessary, it is manipulation.		
34. The quarantine thing is only a suggestion. If I go out alone nothing happens.		
35. The virus will force a debate on authoritarian government systems.	*	
36. We are in 2020 which makes 40 meaning that, from an energetically standpoint, a quarantine was due to trigger a transformation of consciousness.		
37. COVID-19 is fateful. There is a pandemic every century.		
38. COVID-19 has been created as a social experiment to dominate through fear.		
39. COVID-19 has been created as a fearmongering domination experiment by multinationals.	*	*
40. The Annunakis want to spread fear to take control of our minds.		
41. Tracking APPS in Spain would not have the capacity to function efficiently, due to bureaucratic obstacles. Hence, it would be useless.		
42. The coronavirus is the product of the Chinese conspiracy to dominate the world.		
43. Coronavirus tracking APPS are an excuse for a hidden power aiming to control us.		
44. Nature has initiated a change of dimension and needs to expel those who cannot bear it.		
45. In the case of the coronavirus, the WHO lies, as always.		

Phase 1. Exploratory Analysis		Phase 2 Analysis of COVID-19 Implicit Theories (Its) as Knowledge
**Step 1.** ***Historical Review:*** **Items obtained**	**Step 2.** ***Focus group:*** **Items of the Cultural Categories**	**Items of the ITs bout the origin of COVID-19 as knowledge**
46. We are already controlled enough. There is no need to force us to have a coronavirus tracking APP.	*	*
47. The virus is programmed to establish a new world order.		
48. There is evidence that the Annunaki want to control humanity.		
49. The Coronavirus Pandemic is just a smokescreen to distract us from extra-terrestrial control.	*	*
50. They want us to become slaves using the virus as an excuse.		
51. Humanity needs to wake up from the deception of COVID-19.		
52. The planet has increased its level of consciousness and is using the coronavirus to make humanity grow.	*	*
53. We must stop collaborating with the fearmongering that wants to enslave us.		
54. Powerful groups behind the scenes have an unacceptable programmed life in store for us.	*	
55. They want to control us, to program us, that is why they have created the virus.		
56. The Annunaki have released the virus to Earth to spread fear.		
57. COVID-19 does not exist.		
58. There is evidence that aliens have contacted humans to warn them about the COVID-19 lie.		
59. They want to use the vaccine to install a mind control chip to reprogram us.		
60. COVID-19 vaccine is programmed by aliens to subdue us.	*	*
61. Secret powerful groups want to enslave humanity.		
62. People who do not see that COVID-19 is manipulation are blind.		
63. The virus is a fiction. It only exists in your head.		

Phase 1. Exploratory Analysis		Phase 2 Analysis of COVID-19 Implicit Theories (Its) as Knowledge
**Step 1.** ***Historical Review:*** **Items obtained**	**Step 2.** ***Focus group:*** **Items of the Cultural Categories**	**Items of the ITs bout the origin of COVID-19 as knowledge**
64. This is a mental virus imposed by secretive powerful groups.		
65. COVID-19 is a common flu, but scientists and the media have generated fear-mongering propaganda to control us.	*	
66. The planet can no longer endure human aggression and has responded with the virus.		
67. Coronavirus has been caused by nature itself to extinguish humanity.	*	*
68. It was to be expected; the land had to defend itself.		
69. As long as humanity does not respect the planet, pandemics will not stop.		
70. The virus has been created by multinationals to enrich themselves.	*	*
71. A punishment like COVID-19 is what a sick society deserves.		
72. COVID-19 is an opportunity for humanity to improve.		
73. Corrupt politicians have allowed the creation of the coronavirus		
74. Energies from other planets want to take advantage of COVID-19 to enslave humanity.	*	

Note: **Phase 1**. Step 1: A total of 74 items were obtained from the *Historical Review* carried out on social networks and Internet news channels. They were used in the *Focus Group*. Step 2: The *focus group* experts classified a total of 30 items in five *Cultural Categories*. The Implicit Theories Questionnaire about the origin of COVID-19 as Knowledge was elaborated using these items. The remaining items were removed. **Phase 2.** From these 30 items, a total of 20 typical items saturated the four theories of knowledge. The Implicit Theories Questionnaire as Beliefs about the origin of COVID-19 was elaborated using these items and then used in Study 1.

Table_2_SuppInfo

Story-based questionnaire portraying Implicit Theories as Knowledge (Knowledge ITs).

Restrictions of individual freedom Theory

One more day of confinement. This is what **Louise** shares on her Facebook wall: "With each passing day, I am more scared." The experts only strive to find ways to control people."
Immediately, the post gets 15 “likes” and 2 comments:
Peter Pi: Louise, this has only just begun. I have read that they want to implant "microchips" in asymptomatic carriers.
ColonelTal: They are putting one over on us! Bye to free circulation. They will know how long we take in the toilet. 🙁 🙁
==========
Please put yourself in these people’s shoes and respond to the following sentences as they would. Remember, we are not asking for your opinion, we ask you "to become” **Louise**, **Peter Pi** or **ColonelTal** and answer accordingly.

Mother Earth control Theory

One more day of confinement. This is what **Louise** shares on her Facebook wall:
"I am hopeful, as we have ignored the climate or financial crisis, the planet has decided to act and has created a virus that will force us to become aware, finally."
Immediately, the post gets 15 “likes” and 2 comments:
Peter Pi: Louise, you are right! Confinement forces us to become aware of our fear and discover the love that is within us.
ColonelTal: The Earth is intelligent. It does not need us to cleanse itself. We can follow through or not, but the Earth will do what it has to do and it will do it with intelligent love.”
==========
Please put yourself in these people’s shoes and respond to the following sentences as they would. Remember, we are not asking for your opinion, we ask you "to become” **Louise**, **Peter Pi** or **Colonel Tal**" and answer accordingly.

Alien Control Theory

One more day of confinement. This is what **Louise** shares on her Facebook wall:
"Every passing day am more scared. Finally, the extra-terrestrial race that dominates humanity has decided to carry out its hidden intentions”.
Immediately, the post gets 15 “likes” and 2 comments:
Peter Pi: Louise, this has only just begun. They want to enslave us, and they are using fear as an excuse to subjugate us to the safety instructions.
Colonel_Tal: They are putting one over on us! We do not realize that the virus is a plan to subdue us that comes from outside, not from the Earth.
==========
Please put yourself in these people’s shoes and respond to the following sentences as they would. Remember, we are not asking for your opinion, we ask you "to become **Louise**, **Peter Pi** or **Colonel_Tal**" and answer accordingly.

Table_2_SuppInfo (continuation)

Story-based questionnaire portraying Implicit Theories as Knowledge (Knowledge ITs).

**Economic power Control Theory**
One more day of confinement. This is what **Louise** shares on her Facebook wall:<br><br>“I am more and more indignant. I just do not understand this dispute about what comes first, health or economy.<br>*Immediately, the post gets 15 “likes” and 2 comments:*<br>**Peter Pi**: Louise, if the economic powers need a virus to achieve their goals, they will create it.<br>**ColonelTal**: This is a pure horror! In 2008 they created the " financial crisis", in 2020 they have opted for a "viral crisis.<br>==========<br>Please put yourself in these people’s shoes and respond to the following sentences as they would. Remember, we are not asking for your opinion, we ask you "to become” **Louise**, **Peter Pi** or **ColonelTal**" and answer accordingly.
**Social Change Theory**
One more day of confinement. This is what **Louise** shares on her Facebook wall:<br>"Every cloud has a silver lining. All this will force governments and citizens to rethink the current social model".<br>*Immediately, the post gets 15 “likes” and 2 comments:*<br>**Peter Pi**: Louise, you are right! When this is over, the rich will be richer and the poor poorer.<br>**Coronel_Tal**: People must stop and think! This whole coronavirus disaster is shaking the system.<br>==========<br>Please put yourself in the shoes of these people and respond to the following sentences as they would. Remember, we do not ask for your opinion, we ask you "to become **Louise**, **Peter Pi** or **Colonel_Tal**" and answer.

Table_3_SuppInfo

Example of Questionnaire of Implicit Theories as Knowledge (Knowledge ITs).

We are conducting a research on COVID-19 at the University XXX and we would appreciate your participation. Below there is a conversation between three people. Read the conversation carefully, stop for a few seconds and think about how these people perceive the coronavirus crisis. Your task is to answer the sentences that are presented as they would do. **Please, we do not ask for your opinion, we ask you to put yourself in their shoes and answer what they would answer.**

Participation in this research project is voluntary. You can withdraw at any time. The data derived from your participation will be collected and stored in compliance with current data protection regulations and may be used for research, study, and publication purposes, always safeguarding the right to privacy and anonymity, in compliance to Organic Law 15 / 1999, of December 13, Protection of Personal Data (LOPD).

One more day of confinement, **Louise** shares on her Facebook wall: "Each passing day, I am more scared." The experts only strive to find ways to control people. "
Immediately, the post gets 15 "likes" and 2 comments:
Peter Pi: Louise, this has only just begun. I have read that they want to implant "microchips" in asymptomatic carriers.
ColonelTal: They are putting it in us bent! Farewell to circulate freely around there… they will know how long we go to the toilet. 🙁 🙁
==========
Please put yourself in the shoes of these people and respond to the following sentences as they would. Remember**, we do not ask for your opinion, we ask you "to become Louise, Peter Pi or Colonel_Tal" and answer** using the following scale.

**1**<br>**Totally disagree**	**2**<br>**In disagreement**	**3**<br>**Neither agree nor disagree**	**4**<br>**Agree**	**5**<br>**Totally agree**

1. COVID-19's tracking APPS have been created to control people	1	2	3	4	5
2. The planet has created COVID-19 to facilitate the development of a higher state of human consciousness.	1	2	3	4	5
3. Energies from other planets want to take advantage of COVID-19 to enslave humanity.	1	2	3	4	5
4. The coronavirus has been created by laboratories to sell drugs.	1	2	3	4	5
5. The virus will force a debate on authoritarian government systems.	1	2	3	4	5
6. COVID-19 is a common flu, but scientists and the media have generated fear-mongering propaganda to control us.	1	2	3	4	5
7. Coronavirus has been caused by nature itself to extinguish humanity.	1	2	3	4	5
8. On October 11, 2020, a meteorite with COVID-419 particles fell in the area where the pandemic later originated.	1	2	3	4	5
9. The coronavirus was conceived as a selective epidemic devised by capitalism to kill the elderly.	1	2	3	4	5
10. The management of the pandemic will show the inability of current policies to solve problems within the Capitalist Model.	1	2	3	4	5

11. Powerful groups behind the scenes have an unacceptable programmed life in store for us.	1	2	3	4	5
12. The planet has increased its level of consciousness and is using the coronavirus to make humanity grow.	1	2	3	4	5
13. The Coronavirus Pandemic is just a smokescreen to distract us from extraterrestrial control.	1	2	3	4	5
14. COVID-19 is the product of a Chinese conspiracy to have world economic power.	1	2	3	4	5
15. The virus will spotlight current social inequalities on managing the economic crisis.	1	2	3	4	5
16. COVID-19 has been created as a fearmongering domination experiment by multinationals.	1	2	3	4	5
17. Since we have not learned by love, the planet makes us learn by suffering.	1	2	3	4	5
18. The coronavirus tracking APPS have been created by an "Intelligence" from another planet.	1	2	3	4	5
19. COVID-19 has been brought about by aliens through 5G.	1	2	3	4	5
20. The problem is not COVID-19. It is the socio-economic crisis that will come about due to the precariousness of the system	1	2	3	4	5
21. We are already controlled enough. There is no need to force us to have a coronavirus tracking APP.	1	2	3	4	5
22. The Earth has reacted. Humans are destroying it. Hence, it has sent a virus to bring humanity to a standstill.	1	2	3	4	5
23. The coronavirus has an extra-terrestrial origin.	1	2	3	4	5
24. The same person who thinks the earth is overpopulated wants to 'save' your life with a coronavirus vaccine.	1	2	3	4	5
25. The COVID-19 crisis jeopardizes democratic freedoms.	1	2	3	4	5
26. The coronavirus tracking APPS are intended to implicitly violate people's right to privacy.	1	2	3	4	5
27. COVID-19 vaccine will be an opportunity to incorporate a tracking chip to controls us.	1	2	3	4	5
28. COVID-19 vaccine is programmed by aliens to subdue us.	1	2	3	4	5
29. The virus has been created by multinationals to enrich themselves.	1	2	3	4	5
30. The prophecies announced it: confinement due to the pandemic is the path chosen by the planet to transform human consciousness.	1	2	3	4	5

Note: Restrictions of individual freedom Theory: items 1, 6, 11, 21, 25, 26, 27; *Mother Earth control Theory*: items 2, 7, 12, 17, 22, 24, 30;
Economic power control Theory: items 4, 9, 14, 16, 29; *Alien Control Theory*: items 3, 8, 13, 18, 19, 23, 28; *Social Change Theory*: items 5, 10, 15, 20.

Table_4_SuppInfo

Items Typicity and factorial loading of items

Typicity items					Exploratory Factor Analysis			
Items	Alien	Earth	Econo	Freedo	Alien	Earth	Econo	Freed
1. The coronavirus has an extra-terrestrial origin.	**4.55**	1.82	1.48	2.32	**0.94**			
2. COVID-19 vaccine is programmed by aliens to subdue us.	**4.46**	1.64	1.71	2.32	**0.94**			
3. The coronavirus tracking APPS have been created by an "Intelligence" from another planet.	**4.46**	1.82	1.67	2.27	**0.90**			
4. The Coronavirus Pandemic is just a smokescreen to distract us from extra-terrestrial control.	**4.73**	1.59	2.14	2.73	**0.88**			
5. COVID-19 has been brought about by aliens through 5G.	**4.32**	1.86	2.05	3.00	**0.84**			
6. The planet has increased its level of consciousness and is using the coronavirus to make humanity grow.	2.50	**4.18**	2.14	2.05		**0.80**		
7. Since we have not learned by love, the planet makes us learn by suffering.	3.18	**4.27**	1.95	2.32		**0.79**		
8. The planet has created COVID-19 to facilitate the development of a higher state of human consciousness.	3.09	**4.55**	2.10	3.05		**0.76**		
9. Coronavirus has been caused by nature itself to extinguish humanity	3.09	**4.09**	2.10	2.18		**0.71**		
10. The Earth has reacted. Humans are destroying it. Hence, it has sent a virus to bring humanity to a standstill.	2.82	**4.59**	3.14	2.82		**0.64**		
11. The prophecies announced it: confinement due to the pandemic is the path chosen by the planet to transform human consciousness.	3.41	**4.09**	2.19	2.73		**0.63**		
12. The coronavirus has been created by laboratories to sell drugs	3.36	1.73	**4.19**	3.64			**0.82**	
13. The virus has been created by multinationals to enrich themselves.	3.73	2.00	**4.29**	3.59			**0.79**	
14. COVID-19 is the product of a Chinese conspiracy to have world economic power.	3.64	1.64	**4.10**	3.46			**0.77**	
**15.** COVID-19 has been created as a fearmongering domination experiment by multinationals	3.64	2.05	**4.19**	3.64			**0.74**	
**16.** The coronavirus tracking APPS are intended to implicitly violate people's right to privacy.	3.55	3.68	3.43	**4.36**				**0.79**
17. We are already controlled enough. There is no need to force us to have a coronavirus tracking APP.	3.64	2.77	3.33	**4.59**				**0.77**
18. COVID-19 vaccine will be an opportunity to incorporate a tracking chip to controls us.	3.64	2.46	3.24	**4.18**				**0.65**
19. COVID-19’s tracking APPS have been created to control people	3.55	3.32	3.43	**4.09**				**0.62**
20. The COVID-19 crisis jeopardizes democratic freedoms.	3.41	2.86	2.81	**4.68**				**0.51**

Table_5_SuppInfo

Implied Theories of origin COVID-19 Questionnaire (ITs of origin COVID-19)

IT_s of origin COVID-19					
We would like to hear your opinion about COVID-19. Indicate your degree of agreement or disagreement with the following statements.<br>Thank you for your collaboration					
**1<br>Totally disagree**	**2<br>In disagreement**	**3<br>Neither agree nor disagree**	**4<br>Agree**	**5<br>Totally agree**	
1. I believe COVID-19's tracking APPS have been created to control people.	1	2	3	4	5
2. I believe planet has created COVID-19 to facilitate the development of a higher state of human consciousness.	1	2	3	4	5
3. I believe coronavirus has been created by laboratories to sell drugs.	1	2	3	4	5
4. I believe coronavirus has been caused by nature itself to extinguish humanity.	1	2	3	4	5
5. I believe COVID-19 vaccine will be an opportunity to incorporate a tracking chip to controls us.	1	2	3	4	5
6. I believe planet has increased its level of consciousness and is using the coronavirus to make humanity grow.	1	2	3	4	5
7. I believe coronavirus pandemic is just a smokescreen to distract us from extra-terrestrial control.	1	2	3	4	5
8. I believe COVID-19 is the product of a Chinese conspiracy to have world economic power.	1	2	3	4	5
9. I believe COVID-19 has been created as a fearmongering domination experiment by multinationals.	1	2	3	4	5
10. I believe coronavirus tracking APPS have been created by an "Intelligence" from another planet.	1	2	3	4	5
11. I believe COVID-19 has been brought about by aliens through 5G.	1	2	3	4	5
12. I believe we are already controlled enough. There is no need to force us to have a coronavirus tracking APP.	1	2	3	4	5
13. I believe Earth has reacted. Humans are destroying it. Hence, it has sent a virus to bring humanity to a standstill.	1	2	3	4	5
14. I believe coronavirus has an extra-terrestrial origin.	1	2	3	4	5
15. I believe COVID-19 crisis jeopardizes democratic freedoms.	1	2	3	4	5
16. I believe coronavirus tracking APPS are intended to implicitly violate people's right to privacy.	1	2	3	4	5

ITs of origin COVID-19						
We would like to hear your opinion about COVID-19. Indicate your degree of agreement or disagreement with the following statements.<br>Thank you for your collaboration						
**1<br>Totally disagree**	**2<br>In disagreement**	**3<br>Neither agree nor disagree**	**4<br>Agree**	**5<br>Totally agree**		

17. I believe since we have not learned through love, the planet makes us learn by suffering.	1	2	3	4	5
18. I believe COVID-19 vaccine is programmed by aliens to subdue us.	1	2	3	4	5
19. I believe virus has been created by multinationals to enrich themselves.	1	2	3	4	5
20. I believe prophecies announced it: confinement due to the pandemic is the path chosen by the planet to transform human consciousness.	1	2	3	4	5

Note: *Restrictions of individual freedom Theory:* items 1, 5, 12, 15, 16; *Mother Earth control Theory*: items 2, 4, 6, 13, 17, 20; *Economic power control Theory*: items 3, 8, 9, 19; *Alien Control Theory*: items 7, 10, 11, 14, 18.

Table_6_SuppInfo

Study variables

Conspiracy beliefs theories about COVID-19 origin (COVID-19 ITs)

Restrictions of individual freedom Theory

1. I believe COVID-19's tracking APPS have been created to control people.
5. I believe COVID-19vaccine will be an opportunity to incorporate a tracking chip to controls us.
12. I believe we are already controlled enough. There is no need to force us to have a coronavirus tracking APP.
15. I believe COVID-19 crisis jeopardizes democratic freedoms.
16. I believe coronavirus tracking APPS are intended to implicitly violate people's right to privacy.

Mother Earth control Theory

2. I believe planet has created COVID-19 to facilitate the development of a higher state of human consciousness.
4. I believe coronavirus has been caused by nature itself to extinguish humanity.
6. I believe planet has increased its level of consciousness and is using the coronavirus to make humanity grow.
13. I believe Earth has reacted. Humans are destroying it. Hence, it has sent a virus to bring humanity to a standstill.
17. I believe since we have not learned through love, the planet makes us learn by suffering.
20. I believe prophecies announced it: confinement due to the pandemic is the path chosen by the planet to transform human consciousness.

Economic power control Theory

3. I believe coronavirus has been created by laboratories to sell drugs.
8. I believe COVID-19 is the product of a Chinese conspiracy to have world economic power.
9. I believe COVID-19 has been created as a fearmongering domination experiment by multinationals.
19. I believe virus has been created by multinationals to enrich themselves.

Alien Control Theory

7. I believe Coronavirus Pandemic is just a smokescreen to distract us from extra-terrestrial control.
10. I believe coronavirus tracking APPS have been created by an "Intelligence" from another planet.
11. I believe COVID-19 has been brought about by aliens through 5G.G.
14. I believe coronavirus has an extra-terrestrial origin.
18. I believe COVID-19 vaccine is programmed by aliens to subdue us.

Mistrust in Institutional Information (Mistrust)

How do you feel about the information released by the media?

1. I feel invaded in my privacy
2. I feel manipulated
3. I think there are important information that they hide from us
4. I trust the government

Expectations about pandemic consequences (Expectations).

What do you think will happen after COVID-19?

1. Scientific thought will be strengthened
2. The planet degradationeterioration caused byclimate change will stop
3. Significant steps will be taken to articulate social life based on deliberative and cooperative learning within participatory intelligence systems.
4. Compassion and empathy towards people who have been forced to flee their homes because of natural disasters or wars will increase.
5. Our civilization as we know it will collapse
6. The hypervigilance and control systems of the states will increase therefore restricting freedom.
7. Totalitarianism will increase

Willingness to engage in preventive beahviors

Intention to install a COVID-19 tracking APP (APPS)

What are you willing to do about COVID-19?

1. I will use a tracking application to know if I come by someone infected
2. In no case will I agree to use a tracking app to control COVID-19
3. I will only follow the control rules for COVID-19 if I am obliged to
4. The use of the COVID-19 tracking APPS should be mandatory
5. I will use tracking APPS if they guarantee privacy and transparency

Intention to get vaccinated (Vaccine)

What are you willing to do about COVID-19?

1. I will get vaccinated as soon as a vaccine is available
2. I am not going to get vaccinated

Physical distancing from infected people (Distancing)

What do you think about these statements?

1. It is best to avoid people who have tested positive for COVID-19
2. People who have tested positive for COVID-19 are less dangerous than most people assume.
3. I would not like to have as a neighbour a person who has tested positive for COVID-19
4. People who have tested positive for COVID-19 should be isolated from the rest of the community.

Official information on COVID-19

1. COVID-19 is transmitted by small droplets from infected people.
2. COVID-19 is transmitted by small droplets over long distances (more than 50 meters).
3. COVID-19 is transmitted by touching an infected object, if subsequently we touch the T-zone (eyes, nose or mouth).
4. People who test negative in the PCR test cannot infect.
5. People who test positive in the PCR test cannot infect.
6. People who have generated antibodies cannot infect.
7. Using tissues or tissues to cover my mouth when coughing or sneezing prevents the contagion of COVID-19.
8. Wearing masks in public settings prevents COVID-19.
9. Washing your hands frequently prevents COVID-19.
10. Disinfecting objects and surfaces prevents COVID-19.

11. Greater social distance prevents COVID-19.

12. Avoiding to touch your nose, eyes, and mouth prevents COVID-19.

13. COVID is transmitted through the skin.

14. There is no cure for COVID today.

15. All people affected by COVID-19 develop severe symptoms.

16. People with COVID-19 cannot transmit it if they do not have a fever.

17. Children and young people do not need to take measures to prevent COVID-19.

18. Avoiding crowded public places prevents COVID-19.

19. Isolating and treating people with COVID-19 is an effective way to reduce infections.

20. People in contact with other people infected with COVID-19 should also be isolated.

Note: The Official information on COVID-19 can be consulted at https://www.mscbs.gob.es/profesionales/saludPublica/ccayes/alertasActual/nCov-China/ciudadania.htm
